# Antidepressant Use During Pregnancy and Birth Outcomes: Analysis From the UK, Norway, and Sweden

**DOI:** 10.1002/pds.70446

**Published:** 2026-08-12

**Authors:** Florence Z. Martin, Viktor H. Ahlqvist, Paul Madley‐Dowd, Michael Lundberg, Jacqueline M. Cohen, Kari Furu, Dheeraj Rai, Harriet Forbes, Kayleigh Easey, Siri E. Håberg, Gemma C. Sharp, Cecilia Magnusson, Maria C. Magnus

**Affiliations:** ^1^ MRC Integrative Epidemiology Unit University of Bristol Bristol UK; ^2^ Population Health Sciences University of Bristol Bristol UK; ^3^ Centre for Academic Mental Health University of Bristol Bristol UK; ^4^ Centre for Fertility and Health Norwegian Institute for Public Health Oslo Norway; ^5^ Epidemiology and Scientific Strategy IQVIA London UK; ^6^ Department of Biomedicine Aarhus University Aarhus Denmark; ^7^ Institute of Environmental Medicine Karolinska Institutet Solna Sweden; ^8^ NIHR Bristol Biomedical Research Centre University Hospitals Bristol and Weston NHS Foundation Trust and University of Bristol Bristol UK; ^9^ Department of Global Public Health Karolinska Institutet Solna Sweden; ^10^ Department of Chronic Diseases Norwegian Institute for Public Health Oslo Norway; ^11^ Department of Non‐Communicable Disease Epidemiology London School of Hygiene and Tropical Medicine London UK; ^12^ Department of Psychological Science University of Bristol Bristol UK; ^13^ Department of Psychology University of Exeter Exeter UK

## Abstract

**Purpose:**

Although antidepressant use during pregnancy has been linked to adverse birth outcomes such as stillbirth and preterm delivery, existing evidence is conflicting and rarely presents absolute risk estimates. We aimed to assess the association between antidepressant use during pregnancy and birth outcomes, alongside estimates of absolute risk to aid clinical interpretation.

**Methods:**

We conducted a multi‐country cohort study using data from the UK Clinical Practice Research Datalink (1996–2018), Norwegian Medical Birth Registry (2009–2020), and Swedish Medical Birth Register (2006–2020). Discordant sibling analyses and paternal negative controls were used to investigate associations with stillbirth, neonatal death, gestational age, size for gestational age, and Apgar score < 7 at 5‐min among pregnancies delivered at ≥ 22 weeks' gestation.

**Results:**

Among 120 209 (4.8%) pregnancies exposed to antidepressants, maternal use was associated with stillbirth (adjusted pooled odds ratio [aOR] 1.16, 95% CI 1.05–1.28), preterm delivery (aOR 1.26, 95% CI 1.23–1.30), and low Apgar score (aOR 1.83, 95% CI 1.75–1.91). Associations persisted in sibling analyses with greater uncertainty. The predicted absolute risk of stillbirth was 0.34% among unexposed pregnancies and 0.40% among exposed. When restricting to mothers with depression or anxiety, the association with stillbirth attenuated (aOR 1.07, 95% CI 0.94–1.21). Paternal antidepressant use was modestly associated with preterm delivery and low Apgar score.

**Conclusions:**

Maternal antidepressant use during pregnancy may be associated with increased risks of preterm delivery and low Apgar score, though absolute risks remain low. Residual confounding related to parental mental health is likely to explain part of these associations.

## Abstract (highlights)

Key Points

- Current conclusions regarding antidepressant use during pregnancy and birth outcomes are mixed.
- We used three national data sources (UK, Norway, and Sweden) to assess antidepressant use during pregnancy and several birth outcomes using several methodological approaches.
- Antidepressant use during pregnancy was associated with an increased risk of stillbirth, preterm delivery, and low Apgar score; the association with stillbirth attenuated when restricting to those with depression or anxiety.
- Paternal antidepressant use during pregnancy was associated with an increased risk of preterm delivery and low Apgar score.
- Maternal antidepressant use may be associated with a modest increase in risk of preterm delivery and low Apgar score, which is likely partially explained by unmeasured confounding by genetic and environmental factors according to results from the paternal and sibling analyses; modest differences in absolute risk were reassuring for these outcomes compared to unexposed.

## Plain Language Summary

Many people use antidepressants during pregnancy, but research on their effects on babies at birth has produced mixed results and often does not clearly show how common these outcomes are. Using pregnancy and birth records from the UK, Norway, and Sweden, we compared outcomes among babies whose mothers used antidepressants during pregnancy with those who did not. We also used comparisons between siblings and examined antidepressant use in fathers to help account for family and genetic factors.

About 5 in 100 pregnancies involved antidepressant use. Babies exposed during pregnancy were slightly more likely to be born early or to have a low Apgar score shortly after birth. Stillbirth was also slightly more common, but remained rare overall, occurring in about 3 to 4 out of every 1000 unexposed pregnancies and about 4 out of every 1000 exposed pregnancies. When analyses were limited to mothers with depression or anxiety, the link between antidepressant use and stillbirth was weaker.

Overall, while antidepressant use during pregnancy was associated with some increased risks, the absolute risks were low. Some of these findings may reflect factors related to parental mental health rather than the medications themselves, highlighting the need to balance potential risks with the benefits of treating depression and anxiety during pregnancy.

## 1 Purpose

Antidepressant use is rising globally, including during pregnancy [1, 2, 3, 4, 5, 6]. It is estimated that antidepressant prescriptions are made in as many as 8% of pregnancies in the UK [7], 1%–2% in Norway [8, 9], and 3%–4% in Sweden [8]. Antidepressants should not be routinely stopped in pregnancy; treatment decisions in the UK, Sweden, and Norway primarily balance maternal relapse risk against fetal/neonatal risks. SSRIs are commonly used, with drug choice based on prior response, pregnancy stage, and available safety data [10, 11, 12]. The safety profile of antidepressants during pregnancy is difficult to ascertain, particularly because pregnant women are typically not included in randomised controlled trials. Observational studies are therefore the main source of evidence informing decision‐making regarding antidepressant and other medication use during pregnancy. Confounding by indication is a pervasive problem in these studies is setting, as studies suggest that women with untreated depression may have an increased risk of adverse birth outcomes [13]. Systematic reviews of previous observational studies support an increased risk of adverse birth outcomes, including stillbirth (17 studies, pooled odds ratio (OR) 1.19, 95% confidence interval (CI) 1.06–1.34), preterm delivery (13 studies, pooled OR 1.55, 95% CI 1.38–1.74), lower birth weight (20 studies, standardised mean difference (MD) −74 g, 95% CI −117, −31), and low Apgar score at 5‐min (14 studies; standardised MD −0.33, 95% CI −0.47, −0.20) among women who used antidepressants during pregnancy [14, 15]. However, the interpretability of the studies included in the above reviews may still be limited due to confounding and small sample sizes. We aimed to clarify whether maternal use of antidepressants during pregnancy increases the likelihood of certain birth outcomes, including stillbirth, preterm delivery, and low Apgar score. We combined data from the UK, Norway, and Sweden to improve sample size and evaluate drug‐specific effects, as well as leverage a range of methods, including paternal negative controls and discordant siblings, to account for unobserved familial confounding.

## 2 Methods

### 2.1 Study Participants

We used electronic health record (EHR) data from the UK, Norway, and Sweden in the present study. Details of the data sources have been described briefly below but thoroughly elsewhere; [16, 17, 18, 19] data curation and harmonisation for each country are detailed in Sections S1.1–S1.3. We obtained UK data from the Clinical Practice Research Datalink (CPRD) GOLD, which has ~7% population coverage. Those who had been registered with their GP for at least 12 months prior to pregnancy, had linked secondary care data, and experienced a delivery between 1996 and 2018 were identified in the CPRD GOLD Pregnancy Register [20] and were eligible for inclusion. From Norway, all deliveries between 2009 and 2020 registered in the Medical Birth Registry of Norway were eligible. From Sweden, all deliveries between 2006 and 2020 registered in Sweden's Medical Birth Register were eligible. All singleton deliveries born at ≥ 22 completed gestational weeks' that fulfilled the above criteria were included in the study.

This project was approved by CPRD's Independent Scientific Advisory Committee [21_000362] in the UK, the Swedish Ethical Review Authority in Sweden [DNR: 2020‐05516], and the Committee for Medical and Health Research Ethics of South/East Norway [2017/2546/REK sør‐øst A]. Given pseudonymisation performed by all data sources, written consent from individuals was not required for this study.

### 2.2 Exposures

In the UK, prescription data were available based on the prescriptions written by general practitioners, whereas in Norway and Sweden, we used dispensation of prescription drugs from all ambulatory pharmacies recorded in the Norwegian Prescription Database and the Swedish Prescribed Drug Register, respectively (Sections S1.1.1, S1.2.4, S1.3.3). All filled prescriptions for antidepressants were identified using the Anatomical Therapeutic Chemical (ATC) classification (N06A) that refers to antidepressants in Norway and Sweden, and the respective British National Formulary (BNF) codes in the UK (Section S1.4, Table S1). The primary exposure was any maternal antidepressant use during pregnancy proxied by prescriptions (UK) or dispensations (Norway and Sweden). We also defined antidepressant use according to pregnancy trimester (1st trimester < 13 weeks', 2nd trimester 13–26 weeks + 6 days', and 3rd trimester ≥ 27 weeks'). For preterm delivery (< 37 weeks' gestation), those who initiated antidepressants ≥ 37 weeks' were considered “unexposed”. We examined the nine most prescribed antidepressants during the study period (total number of prescriptions for each antidepressant during pregnancy ranked in Sweden, the largest sample): sertraline, citalopram, fluoxetine, escitalopram, venlafaxine, mirtazapine, amitriptyline, paroxetine, duloxetine, ‘other’ (Table S1), and ‘multiple’ (drug switching or concurrent prescriptions for different antidepressants).

We also retrieved corresponding data on paternal use of antidepressants during pregnancy, as a negative control exposure (Norway and Sweden).

### 2.3 Outcomes

Stillbirth was defined as fetal death upon delivery after 22–28 weeks' gestation, [21] the range referring to the changing definition of stillbirth over the study period in Sweden [22]. Neonatal death was defined as death of a live born infant during the first 28 days (0–27) of life [23]. Due to their rarity, these outcomes were omitted from the analysis of specific antidepressants to protect patient anonymity. Preterm delivery was defined as delivery at < 37 weeks' and post‐term delivery was defined as delivery ≥ 42 weeks' gestation. We generated country‐specific percentiles of birthweight, stratified by sex and gestational week. Small for gestational age (SGA) was defined as those with a birthweight in the < 10th percentile [24] and large for gestational age (LGA) was defined as those in the > 90th percentile [25] as per the country‐specific curves. In the Norwegian and Swedish medical birth registries, Apgar score is recoded at 1‐, 5‐, and 10‐min [17, 23]. Apgar score at 5‐min was chosen here, due to its clinical correlation with mortality and cerebral palsy risk [26]. It was binarized into < 7 and ≥ 7, as scores 7–10 are considered reassuring [26]. Information on neonatal death and Apgar score was not available for the UK. In Sweden, there was no paternal linkage to stillborn babies.

### 2.4 Statistical Analysis

We used multivariable logistic regression models to estimate adjusted ORs and absolute (standardised) risks of each outcome in each country separately; analyses of post‐term delivery were restricted to pregnancies that reached 37 weeks' gestation. We replicated these models for trimester‐specific and antidepressant‐specific analyses. Given the varying degrees of data availability in each country, we utilised a country optimised adjustment approach [27] where covariates common to all countries were included in all models and additional covariates were added in a country‐specific manner. We used complete records analysis (CRA); covariates that had > 5% missing data were dropped from the primary adjustment set in each country then added back in sensitivity analysis (Table S2).

Common covariates included maternal age at delivery (< 20, 20–24, 25–29, 30–34, 35–39, 40–44 and ≥ 45), early pregnancy body mass index (BMI; underweight < 18 kg/m^2^, ‘normal’ weight 18–24.9 kg/m^2^, overweight 25–29.9 kg/m^2^, and obese ≥ 30 kg/m^2^), parity (categorical: 0, 1, 2, 3, and ≥ 4), previous stillbirth (binary), anti‐seizure medication and antipsychotic use in the 12 months prior to pregnancy (binary), smoking anytime during pregnancy (binary), and maternal depression or anxiety diagnosis prior to pregnancy start (binary, ≥ 1 code any time during pre‐pregnancy follow‐up). Administrative codes were used to ascertain the presence of a potential indication for antidepressant prescription (evidence of a depression or anxiety diagnosis: ICPC‐2/ICD‐10 codes in Norwegian primary/secondary care, Read codes/ICD‐10 codes in UK primary/secondary care, and ICD‐9/10 codes in Swedish secondary care), and to define prescription/dispensation of anti‐seizure and antipsychotic medications (BNF codes in the UK and ATC codes in Norway and Sweden). All codes are summarised here: https://github.com/flozoemartin/codelists.

We retrieved different proxy measures of socioeconomic position (SEP). This included maternal educational attainment (categorical: compulsory or less, secondary, post‐secondary, post‐graduate, and missing) and country of birth (binarized into foreign‐born or not) in Norway and Sweden, household disposable income according to quintiles in Sweden, and practice‐level Index of Multiple Deprivation (IMD) quintile in the UK. We additionally retrieved ethnicity (White, South Asian, Black, Other, Mixed) [28] for adjustment in the UK. Primary care data were available in the UK and Norway, and the number of primary care consultations in the 12 months prior to pregnancy was extracted for adjustment.

To control for shared genetic and household factors, as well as stable maternal factors between pregnancies, we used a discordant sibling design implemented by conditional logistic regression. Failing to replicate an association within families would suggest that an observed association in the general population might be explained by time‐fixed maternal and household confounders [29]. We also evaluated the association with paternal use of antidepressants while the mother was pregnant (Norway and Sweden only), to clarify the potential the role of residual (household) confounding [30]. We performed mutual adjustment for maternal antidepressant use in sensitivity analysis to limit bias by assortative mating [31]. Adjusted estimates from each country and each methodological approach were then combined using Mantel–Haenszel weighted fixed effects meta‐analysis.

Several sensitivity analyses were performed. We repeated the maternal analysis adjusting for all covariates (irrespective of missing data) and tested for potential bias in the CRA as per Hughes et al. [32] We evaluated the associations with maternal antidepressant use stratified on, as opposed to adjusting for, indication (depression or anxiety) [33]. We stratified preterm delivery according to ‘moderate‐to‐late’ (32–36 weeks + 6 days' gestation), ‘very’ (28–31 weeks + 6 days'), and ‘extremely’ (< 28 weeks') preterm. To explore the role of labour induction, we investigated the risk of pre‐ and post‐term delivery restricted to spontaneous delivery. We re‐ran the analyses for SGA and LGA using definitions as per the INTERGROWTH‐21 [34] and the new Swedish standards [35].

Additional data management decisions were made when cleaning the UK data, so a number of UK‐specific sensitivity analyses were performed, including using alternative sources of information to assign pre‐ and post‐term delivery and changing the threshold of post‐term delivery to reflect the imputation approach leveraged by the authors of the Pregnancy Register algorithm [20] (Section S2.5.1).

All data cleaning, analyses, and visualisations were performed in Stata 17.0; all scripts are open source (https://github.com/flozoemartin/Birth‐outcomes).

## 3 Results

The eligible study population contained 352 361 singleton deliveries from the UK, 679 511 from Norway, and 1 497 044 from Sweden (Figure S1). A total of 120 209 (4.8%) pregnancies were exposed to antidepressants: 6.2%, 2.5%, and 5.4% in the UK, Norway, and Sweden, respectively.

Maternal characteristics were similar across each country (Table 1). An important difference between the countries: prevalence of depression and anxiety was lowest in Sweden, reflecting the lack of primary care data (Table 1). In all three countries, lower SEP (lower educational attainment or higher deprivation quintile) was associated with antidepressant use during pregnancy (Table S3).

**TABLE 1:** Maternal characteristics in eligible populations from each country, stratified by antidepressant exposure status during pregnancy.

Characteristics	UK		Norway		Sweden	
	Exposed to ADs	Unexposed to ADs	Exposed to ADs	Unexposed to ADs	Exposed to ADs	Unexposed to ADs
Total	21 665 (100.0)	330 696 (100.0)	17 202 (100.0)	662 309 (100.0)	81 342 (100.0)	1 415 702 (100.0)
Year of birth						
1996–1999	943 (4.4)	27 790 (8.4)	—	—	—	—
2000–2004	4156 (19.2)	76 139 (23.0)	—	—	—	—
2005–2009^a^	5955 (27.5)	100 196 (30.3)	1331 (7.7)	58 721 (8.9)	11 115 (13.7)	273 287 (19.3)
2010–2015	8174 (37.7)	103 639 (31.3)	8662 (50.4)	340 437 (51.4)	32 284 (39.7)	622 298 (44.0)
2016–2020^b^	2437 (11.2)	22 932 (6.9)	7209 (41.9)	263 151 (39.7)	37 943 (46.6)	520 117 (36.7)
Maternal age at start of pregnancy						
< 20	1270 (5.9)	23 027 (7.0)	302 (1.8)	8985 (1.4)	748 (0.9)	11 990 (0.8)
20–24	4294 (19.8)	53 997 (16.3)	2491 (14.5)	80 952 (12.2)	7605 (9.3)	148 101 (10.5)
25–29	5915 (27.3)	90 987 (27.5)	5150 (29.9)	211 822 (32.0)	21 372 (26.3)	409 082 (28.9)
30–34	5860 (27.0)	101 806 (30.8)	5339 (31.0)	227 919 (34.4)	27 714 (34.1)	499 336 (35.3)
35–39	3377 (15.6)	51 298 (15.5)	3129 (18.2)	109 753 (16.6)	18 450 (22.7)	276 064 (19.5)
40–44	856 (4.0)	9034 (2.7)	740 (4.3)	21 637 (3.3)	5065 (6.2)	65 862 (4.7)
≥ 45	93 (0.4)	547 (0.2)	51 (0.3)	1241 (0.2)	341 (0.4)	4331 (0.3)
Maternal body mass index (BMI)						
Underweight (< 18.5 kg/m^2^)	794 (3.7)	10 782 (3.3)	474 (2.8)	18 173 (2.7)	1576 (1.9)	33 586 (2.4)
Healthy weight (18.5–24.9 kg/m^2^)	8574 (39.6)	157 814 (47.7)	6346 (36.9)	278 924 (42.1)	38 634 (47.5)	780 095 (55.1)
Overweight (25.0–29.9 kg/m^2^)	5263 (24.3)	77 564 (23.5)	3041 (17.7)	101 219 (15.3)	21 129 (26.0)	337 988 (23.9)
Obese (≥ 30.0 kg/m^2^)	5456 (25.2)	54 843 (16.6)	2327 (13.5)	55 787 (8.4)	14 701 (18.1)	176 452 (12.5)
Missing	1578 (7.3)	29 693 (9.0)	5014 (29.1)	208 206 (31.4)	5302 (6.5)	87 581 (6.2)
Maternal smoking during pregnancy						
Smoker	3223 (14.9)	24 851 (7.5)	2729 (15.9)	39 608 (6.0)	10 005 (12.3)	77 271 (5.5)
Missing^c^	0	89 (0.0)	1853 (10.8)	77 717 (11.7)	42 674 (3.3)	44 166 (3.1)
Maternal history of stillbirth at the start of pregnancy						
History of stillbirth	269 (1.2)	2497 (0.8)	142 (0.8)	4810 (0.7)	412 (0.5)	4970 (0.4)
Maternal parity at the start of pregnancy						
0	7853 (36.2)	150 878 (45.6)	7812 (45.4)	280 276 (42.3)	36 662 (45.1)	617 649 (43.6)
1	7622 (35.2)	121 661 (36.8)	5404 (31.4)	244 608 (36.9)	26 453 (32.5)	524 559 (37.1)
2	3907 (18.0)	40 887 (12.4)	2685 (15.6)	98 382 (14.9)	12 406 (15.3)	189 163 (13.4)
3	1514 (7.0)	11 959 (3.6)	876 (5.1)	26 061 (3.9)	3843 (4.7)	52 445 (3.7)
4+	769 (3.5)	5311 (1.6)	425 (2.5)	12 982 (2.0)	752 (0.9)	14 115 (1.0)
Missing	0	0	0	0	1226 (1.5)	17 771 (1.3)
Maternal antidepressant indications ever before pregnancy^d^						
Depression	16 855 (77.8)	66 423 (20.1)	14 165 (82.3)	125 621 (19.0)	32 384 (39.8)	56 272 (4.0)
Anxiety	9862 (45.5)	40 587 (12.3)	11 007 (64.0)	90 715 (13.7)	35 908 (44.1)	91 773 (6.5)
Other mental health‐related prescriptions in 12 months before pregnancy						
Antipsychotics	87 (0.4)	43 (0.0)	1412 (8.2)	5971 (0.9)	2932 (3.6)	4112 (0.3)
Anti‐seizure medications	591 (2.7)	1918 (0.6)	845 (4.9)	3333 (0.5)	2168 (2.7)	5816 (0.4)
Primary care consultations in the 12 months before pregnancy						
0	1168 (5.4)	32 193 (9.7)	260 (1.5)	97 102 (14.7)	—	—
1–3	1420 (6.6)	92 354 (27.9)	1630 (9.5)	210 879 (31.8)	—	—
4–10	7593 (35.0)	144 093 (43.6)	6204 (36.1)	245 999 (37.1)	—	—
> 10	11 484 (53.0)	62 056 (18.8)	9108 (52.9)	108 329 (16.4)	—	—
Initiation of labour						
Induced	7083 (32.7)	87 022 (26.3)	5996 (34.9)	182 931 (27.6)	16 286 (20.0)	225 072 (15.9)
Missing	4696 (21.7)	79 772 (24.1)	0	0	0	0

^a^ Norway study period started in 2009, i.e., births that occurred in 2005–2008 excluded.

^b^ UK study period ended in 2019 i.e., births that occurred in 2020 excluded.

^c^ UK smoking during pregnancy: coded as a smoker if smoking recorded during pregnancy, if not or missing recoded to non‐smoker. Missing smoking data refers to having no matched Clinical or Additional data.

^d^ Read and ICD‐10 codes (UK, primary and secondary care), ICPC2 and ICD‐10 codes (Norway, primary and secondary care), ICD‐9 and ICD‐10 codes (Sweden, secondary care only).

Overall, the rates of most outcomes were similar between countries. Among unexposed pregnancies, 0.3%–0.5% ended in stillbirth, 4.7%–6.6% were born preterm, and 4.5%–6.7% were born post‐term. Among the exposed, 0.4%–0.6% ended in stillbirth, 7.3%–8.6% were preterm, and 3.1%–4.7% were post‐term (Tables S4 and S5).

### 3.1 Maternal Analysis

**FIGURE 1:**
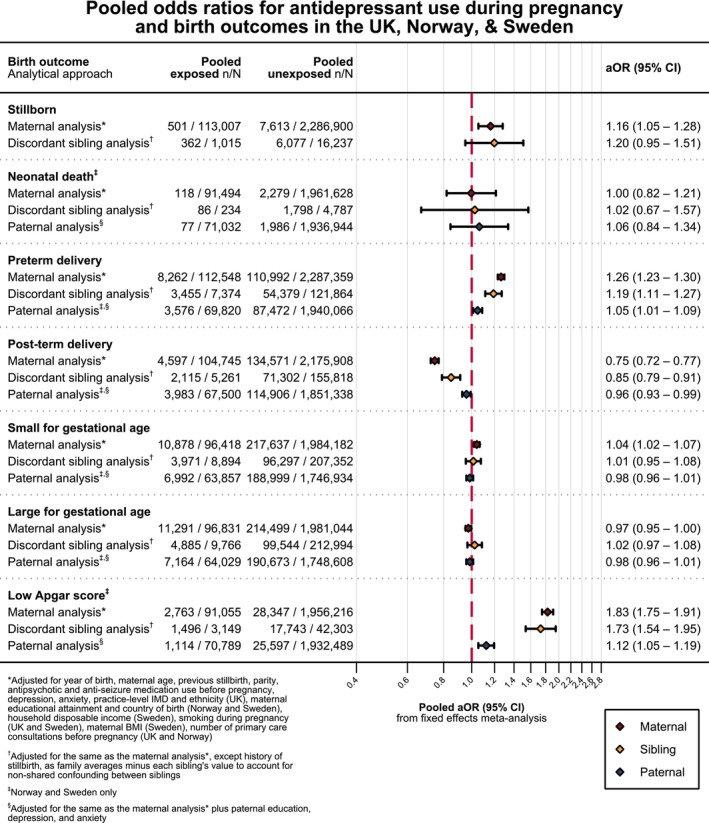
Primary analysis of maternal antidepressant use during pregnancy, maternal antidepressant use among discordant siblings, and paternal antidepressant use during pregnancy as a negative control.

In the fixed effects meta‐analysis of maternal antidepressant use during pregnancy, we observed an increased risk of stillbirth (adjusted OR (aOR) 1.16, 95% CI 1.05–1.28) (Figure 1). The adjusted absolute risk of stillbirth was 0.34% (95% CI 0.33–0.35) in the unexposed and 0.40% (95% CI 0.36–0.44) in the antidepressant exposed (Figure 1). We also observed an increased risk of preterm delivery (aOR 1.26, 95% CI 1.23–1.30), SGA (aOR 1.04, 95% CI 1.02–1.07), and Apgar score < 7 at 5 min (aOR 1.83, 95% CI 1.75–1.91), as well as a decreased risk of post‐term delivery (aOR 0.75, 95% CI 0.72–0.77) and no notable difference in the risk of LGA nor neonatal death (Figure 1). Effect estimates from each country contributing to each analysis can be found in Figure S2.

**FIGURE 2:**
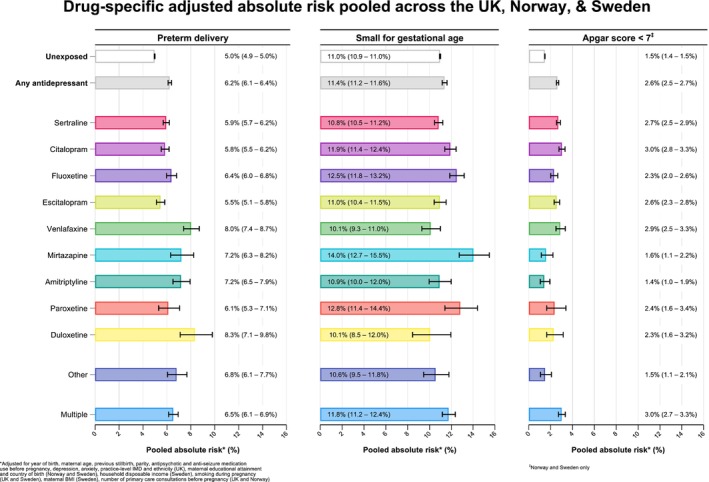
Pooled absolute risk adjusted for confounders of preterm delivery, SGA, and low Apgar score for each antidepressant medication.

**FIGURE 3:**
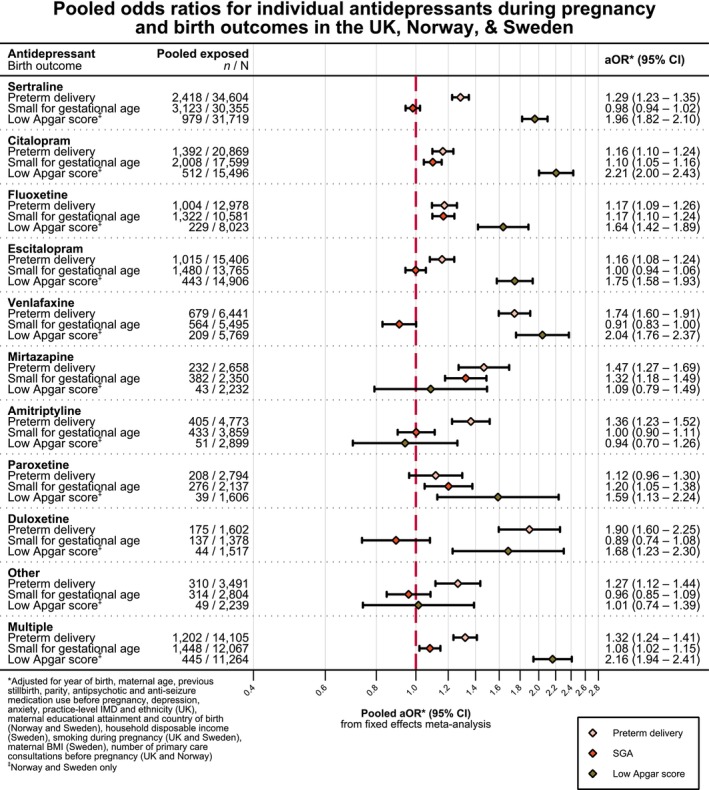
Drug‐specific analyses for preterm delivery, small for gestational age, and Apgar score < 7 at 5 min after delivery.

The trimester‐specific analysis indicated the risk of low Apgar score increased with trimester of exposure and risk of post‐term decreased (Figure S3). Most antidepressants were associated with preterm delivery and Apgar score < 7 at 5‐min (Figure 2, Figure S4, Figure S6). The use of citalopram, fluoxetine, mirtazapine, or paroxetine during pregnancy was also associated with a higher risk of SGA (Figure 3, Figure S5). The increased risk translated to modest differences in absolute risk adjusted for confounders across all medications (Figure 2) and adjusted mean differences in gestational week and weight at delivery (Tables S6 and S7).

### 3.2 Discordant Sibling Analysis

The number of discordant sibling pairs available for the different analyses ranged from 419 to 2743 in the UK, 108 to 2155 in Norway, and 273 to 9794 in Sweden (Table S8). The sibling analyses yielded similar results to the primary analysis, albeit with wider confidence intervals (Figure 1, Figure S7).

### 3.3 Paternal Negative Control Analysis

Information on paternal use of antidepressants was available for 2 127 142 deliveries in Norway and Sweden. Fathers used antidepressants while their partner was pregnant in 76 080 (3.6%) of the deliveries included in the sample. Paternal background characteristics according to use of antidepressants in Norway and Sweden (Table S9). The associations with paternal use of antidepressants were null for most outcomes (Figure 1, Figure S8). The upper CI remained below one for post‐term delivery and the lower CI remained above one for preterm delivery and low Apgar score. Further adjustment for maternal use of antidepressants did not change our conclusions for paternal antidepressant use (Figure S9).

### 3.4 Sensitivity Analyses

We observed similar associations when adding ethnicity and BMI (UK) and smoking (Norway) to the primary analysis model (Figure S10). When restricting the populations to those with evidence of depression or anxiety prior to pregnancy, we observe similar results to the primary analysis, although an attenuation to the null for stillbirth was seen in Sweden (Figure S11). Antidepressant use was associated with increased risk of ‘moderate‐to‐late’, but not ‘very’ or ‘extremely’ preterm delivery (Figure S12), and an increased risk of both spontaneous and induced preterm delivery (Figure S13). When restricting to spontaneous deliveries in the evaluation of the risk of post‐term birth, the reduced risk observed following maternal antidepressant use in the main analysis slightly attenuated (Figure S14). Using the INTERGROWTH‐21 and new Swedish standards (Table S1), we observed similar results to the primary analysis for SGA and LGA (Table S10). The UK‐specific sensitivity analyses are summarised in Tables S11–S13. To note, using different methods to define post‐term birth, including gestational age at birth reported in secondary care or codes that denoted the delivery as ‘late’, we observed findings in the UK that reflected the suggested protective association observed in Norway and Sweden (Table S12).

## 4 Conclusions

In this large study including data from the UK, Norway, and Sweden, we found that women who used antidepressants during pregnancy had an increased risk of stillbirth, preterm delivery, and low Apgar score at 5‐min post‐delivery. The lower risk of post‐term delivery among women who had used antidepressants in the main analysis was not completely explained by induction of labour and warrants further investigation.

In response to previous studies where these analyses were called for, we investigated trimester‐specific exposure [36]. As trimester of use increased, antidepressants appeared more protective for post‐term delivery and LGA, whereas the risk of preterm delivery, SGA, and low Apgar score increased. However, the confidence intervals for the trimester‐specific exposure were mostly overlapping.

All antidepressants were associated with an increased risk of preterm birth (although the estimate for paroxetine was imprecise), and most were associated with a low Apgar score. The associations between individual drugs and SGA were mixed, where citalopram, fluoxetine, mirtazapine, paroxetine, and ‘multiple’ were associated with an increased risk, whereas sertraline, escitalopram, duloxetine, venlafaxine, and ‘other’ were not.

Our findings are in line with existing evidence from meta‐analyses indicating increased risk of stillbirth (0.36% *v* 0.40% in the unexposed and exposed, respectively), preterm birth (5.0% *v* 6.2%), and low Apgar score (1.5% *v* 2.6%) following antidepressant use during pregnancy [14, 15, 37]. The stillbirth finding aligns closely with the systematic review findings from Martin et al. (pooled aOR 1.16 vs. summary effect estimate 1.19 [14]). Some individual studies of stillbirth, however, have concluded that antidepressants are not associated with an increased risk of stillbirth [38, 39]. Specifically, Stephansson et al. showed an attenuation to the null when restricting to those with a previous psychiatric hospitalization [39]. We observed a small but clinically insignificant effect for SGA (11.0% vs. 11.4%) that attenuated in the discordant sibling models, in line with the findings from Sujan et al. [36] and in conflict with others [40, 41]. Our findings are also consistent with previous studies that have reported associations between SSRIs and low Apgar score [15, 42]. SSRIs during pregnancy have been previously associated with delayed neonatal adaptation [41, 43] which includes low Apgar score. We additionally showed an association with venlafaxine and duloxetine (SNRIs) with low Apgar score, but not amitriptyline (TCA) or mirtazapine (atypical). Our finding for preterm delivery is likely partially driving the increased risk of low Apgar score [44]. In spite of this, we reassuringly found no association between antidepressant use during pregnancy and neonatal death.

The results from the sibling analyses indicated that our findings were unlikely to be explained by unmeasured confounding by time‐fixed maternal characteristics. However, confounding by indication cannot be ruled out, as symptoms of depression vary over time. We found mostly null results in the paternal negative exposure control analysis, cautiously reassuring us that shared environment confounding was adequately handled in the maternal analysis.

A biological effect of maternal antidepressant use during pregnancy on these birth outcomes is plausible. Serotonin plays an important role in fetal development [45] and circulating (free) serotonin is higher in those taking antidepressants, particularly those that block serotonin reuptake, like SSRIs [46]. Animal studies have shown high serotonin exposure can lead to placental inflammation [47] which is implicated in stillbirth and preterm delivery [48]. Regarding the antidepressant‐specific analyses, although they are all monoaminergic‐acting, they differ in receptor activity, active metabolites, and placental transfer, for example [49]. However, existing evidence has not established consistent drug‐specific patterns of risk and given that maternal stress also interacts with the serotonergic system, [50] it is difficult to disentangle confounding by indication.

The present study has several strengths. It leveraged data from three countries, two of which (Norway and Sweden) have near full population coverage, speaking to the study's generalisability. The size of these pooled data and within‐family linkage capabilities facilitated the use of paternal negative exposure control analyses (excluding UK) and discordant sibling analyses. To our knowledge, we are the only study that has provided estimates of absolute risk adjusted for confounders alongside the relative. The study also has some limitations. We used a combination of prescriptions written (UK) and dispensed (Norway and Sweden) to assign exposure status. There is likely some non‐differential exposure misclassification if the medication wasn't taken, especially in the UK where medication may not have been dispensed; [51] the impact of this type of misclassification is unclear [52]. The definition of the exposure as “any during pregnancy” does not reflect real‐world prescribing of antidepressants [53] and does not take into consideration things like discontinuation [7, 54]. We attempted to mitigate this in the trimester‐specific analysis, which should be interpreted carefully; increasing risk cross trimesters in the present analysis may represent a higher cumulative exposure by trimester three or exposure in a period of increased vulnerability. Our present approach precludes our ability to rule out either as potential explanations.

The findings from the sibling analysis mostly reflected the findings from the primary maternal analysis. However, these are still sensitive to all confounders that are not shared across the pregnancies, [29] such as any new‐onset disease. We observed a small effect for paternal antidepressant use and both preterm delivery and Apgar score, meaning we cannot rule out residual confounding for these outcomes. We used evidence of a diagnosis of depression or anxiety to proxy potential antidepressant indication, but we don't know from EHR data that any one diagnosis refers to the indication for a medication. Depression and anxiety are conditions with variable clinical trajectories, [55] involving periods of remission and relapse, making it likely that exposure to antidepressants is in part proxying periods of “active” illness and resulting in unmanaged confounding by severity of indication. To note, indication was obtained differently across countries: unlike the UK and Norway, primary care data were not available in Sweden, meaning that potential indication was obtained only for those who had been hospitalised or sought specialist care for depression or anxiety. Confounding by severity of indication is particularly problematic in drug‐specific analyses, where SSRIs are common first line treatments and other classes are prescribed more infrequently. Stronger associations for non‐SSRI medications than SSRIs and for exposure in later trimesters are both compatible with confounding by severity of indication. Prevalence of prescribing across countries differed which may have further compounded confounding by severity of indication. Although a hot‐decking imputation approach was used to estimate prescription length where data were missing in the UK data, no further approaches were used to handle missing data; the use of a CRA may have introduced selection bias to the findings. An additional limitation is that our data only captured pregnancies reaching at least 22 weeks' gestation, meaning our findings represent the direct effect of treatment on the events of interest not mediated through competing events [56]. Early pregnancy loss may act as a competing event for subsequent birth outcomes because fetuses that are lost are no longer at risk of outcomes such as preterm birth. If the exposure influenced the risk of early pregnancy loss, restricting the analysis to pregnancies surviving beyond 22 weeks could introduce selection bias, potentially attenuating or exaggerating associations with later birth outcomes, which we did not account for in the present analysis.

In the analysis of post‐term delivery, the restriction to term pregnancies may have introduced selection bias if antidepressant exposure influences the probability of reaching term. Furthermore, delivery before 42 weeks precludes the occurrence of post‐term delivery and therefore represents a competing event. Consequently, the observed lower risk of post‐term delivery among antidepressant‐exposed pregnancies may reflect a shift toward earlier delivery, rather than a direct effect on the risk of prolonged gestation. However, country‐level differences in post‐term delivery were observed (Figure S2), in spite of a persistent increase in risk of pre‐term delivery across all three geographies, suggesting that other factors such as setting‐specific obstetric care differences may be influencing the effect. Future studies may consider using survival models or other approaches to tease apart these potential drivers of the finding we show here.

We would urge caution in interpreting these findings as causal effects that could be used to inform clinical decision making, given the challenge of extricating the effect of the medication from the total effect of the medication and the indication. The absolute risk of each outcome for pregnancies exposed to antidepressants remains small as compared to unexposed pregnancies and should be used in conjunction with the relative odds when interpreting the findings for clinicians and pregnant individuals. Any decisions regarding antidepressant use during pregnancy should weigh the risk of untreated psychiatric conditions against the increased risk of adverse outcomes found in this study, utilising the estimates in absolute risk. Future studies might implement a maternal negative control or more complex approaches such as target trial emulation where data allows.

In the present study, maternal antidepressant use during pregnancy was associated with a modest increase in risk of preterm delivery and low Apgar score. It is important to temper these findings against the estimated absolute risk for each outcome which remained low.

## Author Contributions

**Florence Z. Martin:** conceptualization; data preparation; data cleaning; formal analysis; investigation; interpretation; visualization; writing original draft; submission to journal. **Viktor H. Ahlqvist:** conceptualization; data preparation; data cleaning; interpretation; and editing. **Paul Madley‐Dowd:** conceptualization; data cleaning; interpretation; and editing. **Jacqueline M. Cohen:** conceptualization; data preparation; interpretation; and editing. **Kari Furu:** conceptualization; data preparation; interpretation; and editing. **Dheeraj Rai:** conceptualization; code list checking; interpretation; and editing. **Michael Lundberg:** data preparation; and data cleaning. **Harriet Forbes:** conceptualization; data cleaning; interpretation; and editing. **Kayleigh Easey:** conceptualization; interpretation; and editing. **Siri E. Håberg:** interpretation; and editing. **Gemma C. Sharp:** conceptualization; interpretation; and editing. **Cecilia Magnusson:** conceptualization; data preparation; interpretation; and editing. **Maria C Magnus:** conceptualization; data preparation; data cleaning; interpretation; and editing.

## Funding

The study was supported by the Research Council of Norway through its Centre of Excellence funding scheme (project number 262700) and the European Research Council through the Horizon 2020 research and innovation program (INFERTILITY, grant number 947684). This study was also partly funded by the Research Council of Norway, project no 273366 (InPreSS). FZM was supported by the Wellcome Trust (Grant ref.: 218495/Z/19/Z). DR and HF acknowledge support from the NIH (1R01NS107607). PMD and DR were supported by the NIHR Biomedical Research Centre at the University of Bristol and University Hospitals Bristol and Weston NHS Foundation Trust (NIHR203315). GCS was supported by an MRC grant (MR/S009310/1). KEE is also financially supported by Bristol City Council ‘Understanding and Evaluating Impact: Family Hubs Perinatal Mental Health and Wellbeing Workstream’ (Grant code G01558). The views expressed in this publication are those of the author(s) and not necessarily those of the NHS, the National Institute for Health Research, MRC, or the Wellcome Trust. FZM, PM‐D, VHA, KEE, GCS, DR, and MCM are members of the UK MRC Integrative Epidemiology Unit, which is funded by the MRC (MC_UU_00011/1, MC_UU_00011/3 and MC_UU_00011/7) and the University of Bristol.

## Ethics Statement

The authors assert that all procedures contributing to this work comply with the ethical standards of the relevant national and institutional committees on human experimentation and with the Helsinki Declaration of 1975, as revised in 2013. All procedures involving human subjects/patients were approved by ISAC [21_000362] in the UK, the Swedish Ethical Review Authority in Sweden [DNR: 2020‐05516], and the Committee for Medical and Health Research Ethics of South/East Norway [2017/2546/REK sør‐øst A]. MRC Integrative Epidemiology Unit, University of Bristol, UK; Population Health Sciences, University of Bristol, UK; Centre for Academic Mental Health, University of Bristol, UK; Centre for Fertility and Health, Norwegian Institute for Public Health, Norway; Department of Biomedicine, Aarhus University, Denmark; Institute of Environmental Medicine, Karolinska Institutet, Sweden; NIHR Bristol Biomedical Research Centre, University Hospitals Bristol and Weston NHS Foundation Trust and University of Bristol, Department of Global Public Health, Karolinska Institutet, Sweden; Department of Chronic Diseases, Norwegian Institute for Public Health, Norway; Department of Non‐communicable Disease Epidemiology, London School of Hygiene and Tropical Medicine, UK; Department of Psychological Science, University of Bristol, UK; Department of Psychology, University of Exeter, UK.

## Conflicts of Interest

The authors declare funding from the Research Council of Norway, the European Research Council, the Wellcome Trust, the National Institute for Health and Care Excellence (NIHR), and the Medical Research Council (MRC) (grant numbers detailed below). These funders played no role in study design, data analysis, interpretation, or preparation of the manuscript. The authors are based across the University of Bristol, the University of Exeter, Karolinska Institutet, and the Norwegian Institute for Public Health. The corresponding author is currently employed by IQVIA but joined the company after completion of the research presented in this manuscript. IQVIA had no role in the study design, data collection, analysis, interpretation, or manuscript preparation. We declare no other conflicts of interest.

## Supporting information


**Table S1:** Antidepressant codes used to define the exposure in the present study.
**Table S2:** Covariates that were included in the primary adjustment set based on a priori knowledge and ≤ 5% missing data.
**Table S3:** Proxy measures of socioeconomic position in each country.
**Table S4:** Outcome prevalence stratified by antidepressant exposure during pregnancy in the UK, Norway, and Sweden.
**Table S5:** Proportion of preterm that fall into moderate‐to‐late, very, and extremely preterm categories.
**Table S6:** Drug‐specific analysis of gestational age.
**Table S7:** Drug‐specific analysis of birthweight and small for gestational age in UK (average birth weight 3330 g).
**Table S8:** Patterns of discordance among siblings in each country for each outcome.
**Table S9:** Maternal and paternal characteristics by paternal exposure status.
**Table S10:** SGA and LGA analyses in UK, Norway, and Sweden using different thresholds: from the INTERGROWTH‐21 standard and the new Swedish standard.
**Table S11:** Primary analysis run in the full sample (eligible Pregnancy Register) for variables that are available in the Pregnancy Register in the UK's CPRD GOLD: stillbirth and gestational age at delivery.
**Table S12:** Pre‐ and post‐term analyses among those flagged as pre‐ or post‐term in the full eligible Pregnancy Register sample.
**Table S13:** Post‐term birth defined as ≥ 41 weeks' gestation at delivery.
**Figure S1:** Flow diagrams of pregnancies through the study in each country: UK, Norway, and Sweden.
**Figure S2:** Individual country and pooled odds ratios from fixed‐effect meta‐analysis of maternal models (any antidepressant use during pregnancy compared with no use) for each outcome in each outcome where available.
**Figure S3:** Individual and pooled odds ratios from fixed effect meta‐analyses of antidepressant use in each trimester in the UK, Norway and Sweden for each outcome.
**Figure S4:** Individual and pooled odds ratios from fixed effect meta‐analyses of individual antidepressants during pregnancy and preterm delivery in the UK, Norway and Sweden for each outcome.
**Figure S5:** Individual and pooled odds ratios from fixed effect meta‐analyses of individual antidepressants during pregnancy and SGA in the UK, Norway and Sweden for each outcome.
**Figure S6:** Individual and pooled odds ratios from fixed effect meta‐analyses of individual antidepressants during pregnancy and SGA in the UK, Norway and Sweden for each outcome.
**Figure S7:** Fixed‐effect meta‐analysis of estimates generated from sibling models (any antidepressant use during pregnancy compared with discordant siblings) for each outcome in each outcome where available.
**Figure S8:** Fixed‐effect meta‐analysis of estimates generated from paternal models (any paternal antidepressant use during pregnancy compared with no use) for each outcome in each outcome where available.
**Figure S9:** Fixed effects meta‐analysis of mutually adjusted paternal models in Norway and Sweden where available.
**Figure S10:** Meta‐analysis of each country for additional adjustment sensitivity analyses.
**Figure S11:** Meta‐analysis of adjusted estimates from each country for indication‐based sample sensitivity analyses.
**Figure S12:** Meta‐analysis of adjusted estimates for each country for stratified preterm delivery analyses: moderate‐to‐late preterm (32–37 weeks' gestation), very preterm (28–32 weeks' gestation), and extremely preterm delivery (< 28 weeks' delivery).
**Figure S13:** Meta‐analysis of adjusted estimates for each country for type of labour initiation among preterm deliveries.
**Figure S14:** Meta‐analysis of adjusted estimates for each country for post‐term delivery sensitivity analysis among spontaneous deliveries.

## Data Availability

The data were made available under each stated project number. Access to CPRD data, including UK Primary Care Data, and linked data such as Hospital Episode Statistics, is subject to protocol approval via CPRD's Research Data Governance (RDG) Process. Data from the Norwegian national health registries are available upon application though an online proposal system. Information regarding access can be found on the Directorate of e‐health website (https://helsedata.no/no/om‐helsedata/). Swedish data may be obtained following approval from Statistics Sweden. For more information visit https://www.scb.se/en/services/guidance‐for‐researchers‐and‐universities/ or contact Statistics Sweden at mikrodata@scb.se. Analytic code availability: All analytical code is available on GitHub: https://github.com/flozoemartin/Birth‐outcomes.
